# Deciphering the intrinsically disordered characteristics of the FG-Nups through the lens of polymer physics

**DOI:** 10.1080/19491034.2024.2399247

**Published:** 2024-09-16

**Authors:** Atsushi Matsuda, Abdullah Mansour, Mohammad R. K. Mofrad

**Affiliations:** a Molecular Cell Biomechanics Laboratory, Departments of Bioengineering and Mechanical Engineering, University of California Berkeley, Berkeley, CA, USA; b Molecular Biophysics and Integrative Bioimaging Division, Lawrence Berkeley National Laboratory, Berkeley, CA, USA

**Keywords:** FG-Nups, intrinsically disordered proteins, nuclear pore complex, polymer physics, selective permeability

## Abstract

The nuclear pore complex (NPC) is a critical gateway regulating molecular transport between the nucleus and cytoplasm. It allows small molecules to pass freely, while larger molecules require nuclear transport receptors to traverse the barrier. This selective permeability is maintained by phenylalanine-glycine-rich nucleoporins (FG-Nups), intrinsically disordered proteins that fill the NPC’s central channel. The disordered and flexible nature of FG-Nups complicates their spatial characterization with conventional structural biology techniques. To address this challenge, polymer physics offers a valuable framework for describing FG-Nup behavior, reducing their complex structures to a few key parameters. In this review, we explore how polymer physics models FG-Nups using these parameters and discuss experimental efforts to quantify them in various contexts, providing insights into the conformational properties of FG-Nups.

## Introduction

**Figure 1.**
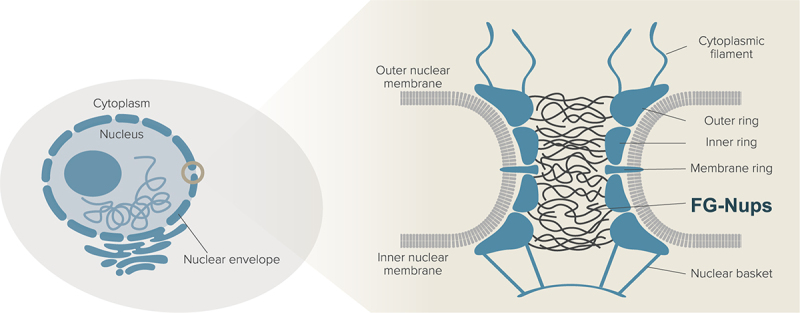
Illustration of the nuclear pore complex (NPC) and FG-Nups. The NPC is a gateway connecting the nucleus and the cytoplasm. The FG-Nups are intrinsically disordered proteins existing within the central channel of the NPC, also extending toward the cytoplasmic filaments and nuclear basket.

The nuclear pore complex (NPC) is a giant protein assembly essential for the eukaryotic cells [1–3]. Embedded within the nuclear envelope, the NPC forms an intricate cylindrical conduit that fuses the inner and outer nuclear membranes (Figure 1). The primary role of the NPC is to regulate the molecular transport across the nuclear envelope. While small molecules, typically those below a threshold of 40 kDa in molecular mass and 5–9 nm in Stokes diameter, can pass through the NPC unimpeded, larger molecules encounter transport barrier unless they are bound to the nuclear transport receptors (NTRs) [4,5]. This selective transport mechanism is crucial for maintaining the cellular integrity and functionality.

The NPC is composed of about 1000 pieces of proteins, making it the largest protein assembly within the cell [6]. It has an outer diameter of 70–120 nm, an inner diameter of 30–60 nm, and a height of 30–50 nm [4,5]. Its vast size and complexity pose significant challenges in unraveling its three-dimensional structure. Over the past decade, a comprehensive approach has been adopted to tackle this challenge, integrating techniques such as X-ray crystallography, cryo-electron tomography, biochemical reconstitution, mass spectrometry, and the application of artificial intelligence. This concerted effort has recently borne fruit, revealing the scaffold structure of the NPC with a remarkable clarity and resolution [7–12].

In contrast to the well-defined scaffold, the configurations of FG-Nups (phenylalanine- and glycine-rich nucleoporins) remain poorly characterized [13–15]. FG-Nups are intrinsically disordered proteins anchored to the inner wall of the central channel. Characterized by their unique residue patterning, i.e. multiple FG-motifs (short, repetitive sequences of phenylalanine and glycine) interspersed with hydrophilic spacer residues, FG-Nups are endowed with significant degrees of conformational flexibility. There are more than 100 FG-Nups within the NPC, which collectively create a dynamic, cloud-like entity spanning the entire channel. Due to their unfolded and dynamic quality of FG-Nups, their structural properties are not captured by the techniques used for resolving scaffold proteins. Nevertheless, comprehending the structural feature of FG-Nups is essential as they play a crucial role in regulating the selective molecular transport through the NPC [16–18].

Considering the intrinsically disordered nature of FG-Nups, analyzing them through the lens of polymer physics presents a viable strategy. Polymers, defined by their long, repeating chains of molecular subunits, are a major focus within theoretical soft matter physics [19]. This branch of physics has significantly contributed to our understanding of macromolecular chemistry, leading to the development of various practical synthetic materials. Given the structural similarities between FG-Nups and polymers, particularly in their repetitive sequences and flexible nature, it’s logical to apply polymer physics principles to study FG-Nups. Indeed, the past decade has seen a dedicated effort to explore the polymer attributes of FG-Nups, which have significantly enhanced our understanding into them [20].

**Figure 2.**
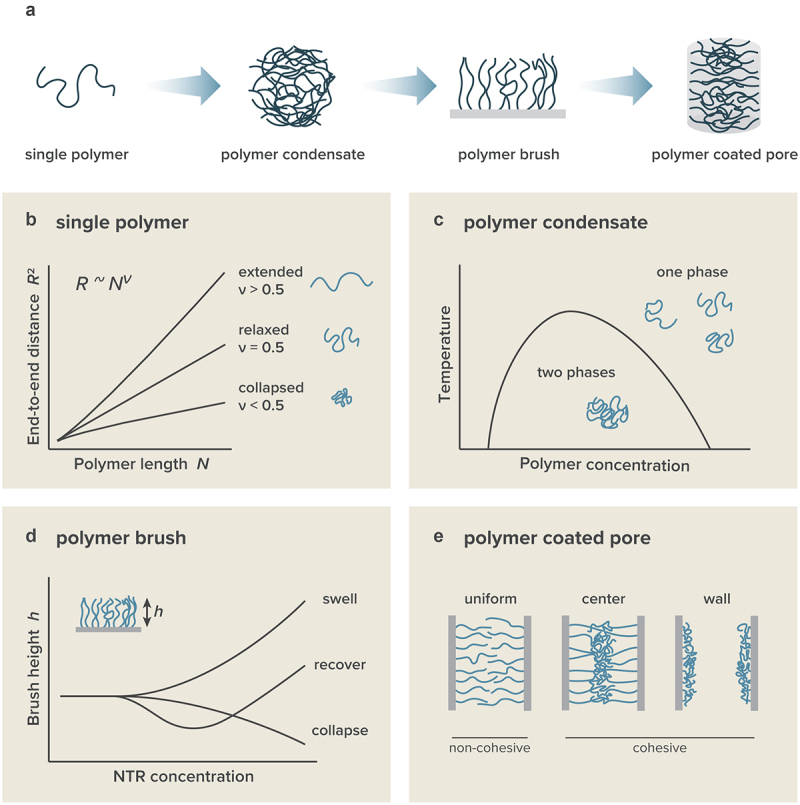
Characteristics of polymers across four different scales. (a) Illustration of the four scales: single polymer, polymer condensate, polymer brush, and polymer coated pore. (b) Single polymer characteristics. Based on the scaling relation, a polymer can be categorized as extended, relaxed, or collapsed. (c) Polymer solution characteristics. Cohesive polymers exhibit phase separation when both polymer concentration and temperature are within certain ranges. (d) Polymer brush characteristics. The addition of NTR results in swelling, recovery, or collapse or the polymer brush. (e) Polymer coated pore characteristics. Non-cohesive polymers display a uniform distribution inside the pore, whereas cohesive polymers tend to aggregate either at the pore’s center or inner walls.

In this review, we present an overview of our current understanding of FG-Nups from the perspective of polymer physics. The review is organized into several sections, each dedicated to examining FG-Nups as single polymers, polymer condensates, polymer brushes, and polymer coated pores (Figure 2). Within each section, we start by outlining the general principles of homopolymer theory, which deals with chains made up entirely of one type of monomer, before correlating these principles with the properties of FG-Nups. Despite the diverse composition of FG-Nups, the application of homopolymer theory often proves to be remarkably effective. In the last section, we reviewed the interactions between FG-Nups and NTRs, a crucial aspect of selective transport mechanisms.

## Single polymer characteristics

In polymer physics, the behavior of a single homopolymer is determined by its flexibility, molecular length, and degree of compaction in a solvent (Figure 2b). For example, the end-to-end distance of the polymer, R, is described by the equation [19]:

(1) $$R\approx bN^{\nu },$$

where b is the Kuhn length, N is the number of segments, and ν is the Flory exponent. The Kuhn length b, defined as twice the persistence length, serves as a measure of the distance over which a polymer chain maintains a straight course along its contour. This parameter indicates the polymer’s flexibility, offering insight into the intrinsic stiffness or pliability of the structure. By discretizing the polymer into segments based on the Kuhn length, the polymer can be conceptualized as a series of N consecutive segments. The relationship between the end-to-end distance R and the number of segments N is governed by the scaling exponent ν. In the case of ideal chains, where there are no interactions among the segments, the polymer’s conformation resembles that of a random walk with step length b, leading to a value of ν=1/2 (theta solvent). However, real chains experience steric repulsion among the segments, causing the polymer to swell and the Flory exponent to increase beyond 1/2 (good solvent). Conversely, if the segments attract each other, the polymer tends toward compaction, resulting in a Flory exponent less than 1/2 (poor solvent).

In exploring the characteristics of FG-Nups within the framework of the homopolymer theory, a critical initial step is to determine their Kuhn length, b. This parameter was effectively estimated by Lim et al. [21,22] through an examination of the force-extension behavior of Nup153. Utilizing atomic force microscopy, they were able to gather data for the force-extension curve of this nucleoporin. By applying the worm-like chain model to their data, they estimated the Kuhn length to be 0.78±0.28 nm [21] and 0.86±0.36 nm [22] in separate studies (corresponding persistence lengths are 0.39±0.14 nm and 0.43±0.18 nm). These estimated Kuhn lengths are notably small, especially when compared to the length of a single amino acid’s peptide backbone, which typically ranges from 0.3 to 0.4 nm. This observation underscores the significant flexibility of FG-Nups, essential feature as intrinsically disordered proteins.

The investigation into the spatial compaction and expansion of yeast FG-Nups has been thoroughly conducted through experiments and simulations (Table 1). The studies measured the hydrodynamic radius of FG-Nups utilizing size-exclusion chromatography [23] and coarse-grained molecular dynamics simulations [24,25]. These measurements were then compared to the hydrodynamic radius of ideal chains, also known as relaxed coils, to categorize the conformation of FG-Nups into three distinct types: collapsed (equivalent to ν<0.5), relaxed (equivalent to ν=0.5), and extended (equivalent to ν > 0.5). The methodology for calculating the hydrodynamic radius of ideal chains draws upon the scaling relation modified for empirical data, as proposed by Tcherkasskaya et al. [2]. This comprehensive analysis revealed that FG-Nups adopt a variety of conformations, significantly influenced by the amino acid composition within the polymer. It was found that a higher ratio of charged to hydrophobic residues tends to promote the extension of FG-Nups, whereas a lower ratio favors a collapsed conformation.

**Table 1.** Single polymer characteristics of yeast FG-Nups. Listed here are FG-Nup names, amino acid sequence ranges (AAs), normalized hydrodynamic radius ($R/R^{*}$), and polymer state. Hydrodynamic radius measurements were obtained from studies employing size-exclusion chromatography (SEC) [23] and molecular dynamics (MD) simulations [24,25]. The reference radius $R^{*}$ is taken from [23], which is the estimation of the ideal chain hydrodynamic radius. Polymer states – collapsed ($R/R^{*}<0.9$), relaxed ($0.9<R/R^{*}<1.1$), and extended ($1.1<R/R^{*}$) – are classified according to their $R/R^{*}$ ratio.

FG-Nup	AAs	$R/R^{*}$			State
		SEC [23]	MD [24]	MD [25]	
Nup116	165-715	0.79	0.78	0.78	collapsed
	765-960	1.00	1.09	1.09	relaxed
Nup100	2-610	0.77	0.78	0.75	collapsed
	611-800	0.96	1.06	1.05	relaxed
Nup145N	1-242	0.69	0.79	0.74	collapsed
	243-433	0.78	0.95	0.95	relaxed/collapsed
Nup49	1-215	0.73	0.89	0.82	collapsed
Nup57	1-255	0.78	0.85	0.83	collapsed
Nup42	1-212	0.77	0.79	0.75	collapsed
Nup1	220-797	1.07	1.08	1.06	relaxed
	798-1076	0.75	0.83	0.75	collapsed
Nsp1	1-172	0.80	0.96	0.86	collapsed/relaxed
	173-603	1.21	1.19	1.17	extended
Nup159	441-881	1.03	1.10	1.07	relaxed/extended
Nup60	389-539	0.93	1.01	1.02	relaxed
Nup2	186-561	1.17	1.06	1.02	relaxed/extended

Further data on the single polymer conformation of FG-Nups is summarized in Table 2. Raveh et al. [26] employed the Anton supercomputer for molecular dynamics simulations on model proteins $\mathrm{FSF}G_{2}$ and $\mathrm{FSF}G_{6}$. These models are constructed from the segments of Nsp1 (AAs 274–397), with minor modifications added. The sequences include 2 and 6 FSFG motifs, respectively, interspaced with 10–30 spacer residues. The study revealed that the conformational state of FG-Nups is highly sensitive to the solvent environment; the use of the TIP4P-D water model yielded a relaxed conformation, whereas the TIP4P-Ew model led to a collapsed state.

**Table 2.** Single polymer characteristics of yeast and human FG-Nups. Listed here are FG-Nup names, amino acid sequence ranges (AAs), solvent environment, Flory exponent (ν), normalized hydrodynamic radius ($R/R^{*}$), polymer state, methodology used, and the reference.

FG-Nup	AAs	Environment	ν	$R/R^{*}$	State	Method	Ref.
Nsp1 ($\mathrm{FSF}G_{6}$)	274–397	TIP4P-D	0.51	-	relaxed	MD	[26]
Nsp1 ($\mathrm{FSF}G_{6}$)	274–397	TIP4P-Ew	0.34	-	collapsed	MD	[26]
Nup153	875-1475	denatured	-	0.99	relaxed	FRET	[27]
Nup153	875-1475	native	-	0.79	collapsed	FRET	[27]
Nup98	1-505	bulk solution	0.29	-	collapsed	FRET	[28]
Nup98	1-505	condensate	0.56	-	extended	FRET	[28]
Nup98	1-505	within NPC	0.56	-	extended	FRET	[28]

The influence of the solvent environment on polymer conformation is further corroborated by experimental studies. Milles et al. [27] employed single-molecule Förster resonance energy transfer (smFRET) to ascertain the end-to-end distance of Nup153 segments, comparing them against those expected from a relaxed coil. Their observations revealed a distinct sensitivity to solvent conditions: under denaturing conditions with 2 M urea, the polymers exhibited relaxed conformations, whereas native conditions resulted in collapsed structures. In a more recent investigation by the same research group [28,29], they studied Nup98 by utilizing a combination of fluorescence lifetime imaging microscopy (FLIM) and FRET. They assessed the polymer conformation across different environments: in bulk solution, phase-separated condensates, and inside a functional NPC. While Nup98 exhibited a collapsed conformation in a bulk solution, it adopted an extended conformation within the condensates and the NPC, showing the complex interplay between the polymer structure and its surrounding environment.

Despite the wide variations in the Flory exponent observed for different types of FG-Nups, Davis et al. suggested that their average behavior closely aligns with that of an ideal chain, i.e., ν=0.5 [30], They first computationally represented FG-Nups 118 as a series of beads, incorporating both excluded volume effects and intermolecular 119 cohesion between the beads. By integrating experimental data from Yamada et al. [23], Denning et al. [31], Kapinos et al. [32] and Wagner et al. [33], they fine-tuned the parameters related to bead cohesion. The results demonstrated that the average behavior of FG-Nups aligns closely with that of ideal chains, where repulsive and attractive interactions are effectively balanced.

## Polymer solution characteristics

In systems containing multiple polymers (polymer solutions), they exhibit collective physical properties, such as phase separation [32,33] (Figure 2c). According to homopolymer theory, the behavior of a polymer solution is defined by two key parameters: polymer concentration, ϕ, and the Flory interaction parameter, χ [19]. The Flory interaction parameter, χ, serves as a dimensionless indicator of the extent to which polymer-polymer interactions surpass polymer-solvent interactions. A positive value (χ>0) indicates a preference for polymers to interact with each other rather than with the solvent, while a negative value (χ<0) suggests that polymers are more inclined to mix with the solvent. The Flory interaction parameter is empirically expressed as a function of temperature, T, in the form χ(T)=A+B/T. The free energy of mixing between the polymer and the solvent (Flory-Huggins-free energy) is represented as [Rubinsten and Colby (2003)]:

(2) $$F_{\mathrm{mix}}=k_{B}T\left[\frac{\phi }{N}\ln\phi +(1-\phi )\ln(1-\phi )+\chi \phi (1-\phi )\right],$$

where N denotes the number of segments in a polymer. The first and second terms represent the entropy contribution from the polymers and solvent, respectively. The third term accounts for the interaction energy between the polymer and the solvent. The stability of the mixing process is determined by the convexity of the free energy. While $\partial^{2}F_{\mathrm{mix}}/\partial \phi^{2}>0$ indicates that the mixing is locally stable, $\partial^{2}F_{\mathrm{mix}}/\partial \phi^{2}<0$ indicates that the mixing is unstable, leading to the phase separation. This relationship forms the basis of the phase diagram for the polymer solution, illustrating the impact of ϕ and χ on phase separation.

The phase separation of the yeast FG-Nups was comprehensively studied by Dekker et al. [25]. Utilizing coarse-grained molecular dynamics simulations, their research focused on determining whether a polymer solution consisting of individual FG-Nups undergoes phase separation. Despite the simulations being performed on FG-Nups having the heterogeneous sequences, the resultant phase diagrams exhibited behaviors consistent with the homopolymers theory. Their results are summarized in Table 3. The study revealed that FG-Nups containing GLFG motifs undergo phase separation, whereas FG-Nups with FxFG motifs do not. They proposed that the observed differences are primarily attributed to the density of FG motifs within the sequences and the ratio of charge to hydrophobicity in the spacer sequences. For comparison, the experimentally measured affinity of FG-Nups as documented by Patel et al. [34] is presented in the same table. This group utilized fluorescently labeled FG-Nups mixed with bead-immobilized FG-Nups to characterize low-affinity protein interactions. Their observations closely align with the simulation results, indicating that FG-Nups demonstrating strong affinity are more likely to undergo phase separation.

**Table 3.** Phase separation of yeast FG-Nups. This table combines the simulated phase separation behavior of various FG-Nups, as conducted by Dekker et al. [25], with the experimentally measured affinities of FG-Nups reported by Patel et al. [34]. AAs denotes the amino acid sequence range of each FG-Nup.

FG-Nup	Dekker et al. [25]		Patel et al. [34]	
	AAs	Phase separation	AAs	Affinity
Nup116	1-725	yes	165–716	++++
	1-965	yes	-	-
Nup100	1-580	yes	1–640	++++
	1-815	yes	-	-
Nup145N	1-219	yes	-	-
	1-458	no	-	-
Nup49	1-246	yes	-	-
Nup57	1-223	yes	1–255	++
Nup42	1-371	yes	29–129	++
Nup159	441–876	no	441-876	no
Nup1	312–1076	no	332–1076	no
	798–1076	yes	-	-
Nsp1	1–601	no	1–603	no
	1–186	yes	-	-
Nup2	160–583	no	181–537	+
Nup60	1–539	no	-	-

The phase separation has also been experimentally demonstrated. Schmidt et al. [35] reported that dispersing small quantities of Nup98 FG domains in aqueous solution caused them to rapidly self-assemble into nearly spherical condensates. Further studies by the same group [36–38] delved into various thermodynamic aspects of these condensates, revealing that the phase separation falls under the category of LCST (lower critical solution temperature) type. Celetti et al. [39,40] documented the phase separation of Nup49 using a custom microfluidic device designed to capture the transient liquid-like state of FG-Nup condensates before they transition into a more solid hydrogel form. Both studies demonstrated that the phase-separated FG-Nups condensates exhibit characteristics that resemble the permeability barrier of the NPC.

## Polymer brush characteristics

In the NPC, FG-Nups are anchored to the inner wall of the channel, which imposes limitations on their structural configurations (Figure 2d). The study of polymer physics frequently involves polymers with ends grafted onto a rigid surface. Particularly, when the grafting distance between polymers is shorter than their radius of gyration, such arrangements are termed ‘polymer brushes’ [19]. These polymer brushes extend in a direction perpendicular to the rigid surface, and their height becomes a critical observable parameter that reflects various physical properties, including the cohesiveness among polymers. The measurement of polymer brush height is conducted using experimental techniques such as atomic force microscopy (AFM), quartz-crystal microbalance with dissipation monitoring (QCM-D), spectroscopic ellipsometry (SE), and surface plasmon resonance (SPR).

For homopolymers, the height of the polymer brush, h, is related to the grafting distance, g, by the scaling relation, $h∼g^{(\nu -1)/\nu }$, s described by the Alexander – de Gennes brush model [19]. Here, ν represents the Flory exponent, defined in Eq. 1. This relationship indicates that measuring the height of the polymer brush allows for the estimation of the Flory exponent, thus facilitating the assessment of polymer cohesiveness. Despite the amino acid sequence heterogeneity in FG-Nups, research by Kapinos et al. [41] and Wagner et al. [42] demonstrated that this scaling relation remains valid for FG-Nups. Their findings reveal that the Flory exponent for FG-Nups ranges widely between ν=1/3 (poor solvent) and ν=3/5 (good solvent), reflecting the diverse cohesiveness levels among FG-Nups. Importantly, the specific amino acid composition of each FG-Nup critically influences its cohesiveness: Eisele et al. [43] demonstrated that the F→S mutation in Nsp1 leads to an effective increase in polymer brush height, highlighting the crucial role of hydrophobic interactions between phenylalanine residues in enhancing cohesiveness.

In the context of the NPC research, the impact of nuclear transport receptors (NTRs) on FG-Nups brushes is a topic of keen interest as highlighted in Table 4. Introducing NTRs into the solution facilitates their interaction with FG-Nups, leading to their penetration into the polymer brush and consequent changes in the brush height. Experimental observations have revealed varied responses of the polymer brush’s height to NTRs: some studies report a significant compaction of the FG-Nups brush upon adding NTRs, whereas others have found that the brush experiences swelling. Furthermore, intermediate behaviors have been recorded where at lower NTR concentrations, the brush collapses, but increasing the concentration leads to a reversal of this compaction, ultimately resulting in the swelling of the brush.

**Table 4.** Change in height of FG-Nups polymer brush upon NTR addition. Shown here is FG-Nup identifier, amino acid sequence range (AAs), NTR type, FG-Nups grafting distance (g in nm), NTR concentration ($c_{\mathrm{NTR}}$ i μM), method for determining polymer brush height, polymer brush behavior, and references. Behavior is categorized as: ‘collapse’ (height decreases), ‘swell’ (height increases), and ‘recover’ (height initially decrease, then increase) in response to rising NTR concentration.

FG-Nup	AAs	NTR	g (nm)	$c_{\mathrm{NTR}}$ (μM)	Method	Behavior	Ref.
Nup62	1–240	Kap1	-	0-0.033	AFM	collapse	[22]
		Kap1	2-11	0.0001-13.4	SPR	recover	[44]
Nup214	1809–2090	Kap1	-	0.0001-13.6	SPR	swell	[41]
Nup62	1–240	Kap1	2.5	0.0001-13.6	SPR	recover	[41]
Nup98	1–498	Kap1	4.0	0.0001-13.6	SPR	recover	[41]
Nup153	874–1475	Kap1	4.5	0.0001-13.6	SPR	recover	[41]
Nsp1	262–492	Kap1	3.7	0.0001-10	SPR	recover	[42]
Nsp1	262–492	NTF2	3.7	0.001-300	SPR	collapse	[42]
Nsp1	2-601	Kap95p	4.4	0.01-5.0	AFM/QCM	swell	[45]
Nsp1	2-601	Kap95p	3.8-6.4	0.01-10	SE/QCM	swell	[46]
Nsp1	2-601	NTF2	3.8-6.4	0.01-10	SE/QCM	swell	[46]
Nup98-glyco	1-485	NTF2	3.8-6.4	0.01-10	SE/QCM	swell	[46]
reg-FSFG	-	NTF2	3.8-6.4	0.01-10	SE/QCM	swell	[46]

The theoretical analysis by Vovk et al. [47] presents a comprehensive explanation for the polymer brush’s diverse reaction to NTRs, resolving the apparent discrepancies observed in experiments. They modeled the free energy of the system in which polymer brush and NTRs coexist. By finding the value minimizing the free energy, they calculated the equilibrium height of the polymer brush. Their result indicates that the height of the polymer brush is determined by multiple parameters, including the grafting density, NTR concentration, the interaction strength between FG-nups and NTRs, the cohesiveness of FG-nups, and the size of the NTRs. The study categorizes the polymer brush’s reaction to NTRs into three distinct behaviors: collapse only, collapse followed by swelling, and exclusive swelling. This categorization allowed them to construct a phase diagram illustrating these behaviors. For instance, they observed that, given the same grafting density and interaction strength, smaller NTRs exhibited a more pronounced collapse than larger NTRs, aligning with experimental observations made by Wagner et al. [42]. This phenomenon is explained by the reduced entropic barrier that smaller NTRs face when penetrating the polymer brush. The phase diagram introduced by them is promising in its potential to explain other experimental findings.

Further exploring the behavior of the polymer brush, Davis et al. [48] investigated the effects of introducing more than two NTRs into the system employing computer simulations. Utilizing density functional theory (DFT), they developed a model to determine the equilibrium density distribution of FG-Nups and two distinct types of NTRs within the polymer brush system. Their findings indicate that altering the concentration of one type of NTR affects the penetration depth of the other type, suggesting that NTRs may play a role in modulating the transport path within the NPC. Additionally, their research revealed that two different NTRs tend to segregate within the polymer brush, with smaller NTRs penetrating deeper and larger NTRs remaining closer to the surface of the brush.

## Polymer coated pore characteristics

In the NPC, FG-Nups are confined within the cylindrical pore, imposing an additional constraint on their behavior (Figure 2e). Unlike a polymer brush on a flat surface, confined FG-Nups cannot extend beyond the pore diameter, leading to crowding that exceeds the equilibrium concentration. Moreover, being tethered on a curved surface causes polymers on one side of the pore to interact with those on the opposite side, complicating their spatial arrangement. Recent studies have highlighted the role of pore dilation in nucleocytoplasmic molecular transport [49–51] underscoring the critical influence of pore geometry on the organization of FG-Nups.

The spacial organization of homopolymers within the pore geometry was computationally studied by Peleg et al. [52]. They utilized molecular theory, i.e. mean field theory for sticky polymers, to calculate the equilibrium distribution of polymers, assessing the effects of various parameters such as pore radius/height, polymer length, polymer cohesiveness, and grafting density. Their findings indicate that in good solvents, polymers stretch out, resulting in a uniform and low-density distribution within the pore. In poor solvents, polymers tend to aggregate, clustering either near the walls to which they are tethered or around the pore’s center. This aggregation behavior is determined by the relationship between the pore radius and the polymer length. Comparable insights were provided by Osmanovic et al. [53], who applied a particle-based model to analyze the homopolymer distribution. They demonstrated that, given a constant pore radius, the location of the polymer aggregate, whether near the pore walls or at its center, is governed by the strength and cutoff distance of the polymer-polymer interactions.

Incorporating the heterogeneity of amino acid sequences into the analysis, the distribution of yeast FG-Nups was examined by three distinct groups, as summarized in Table 5. Utilizing coarse-grained molecular dynamics simulations with slight model adjustments, these studies investigated the spatial organization of FG-Nups within the pore. The outcomes reveal three primary distribution patterns: ‘wall’ for aggregation near the pore walls, ‘center’ for aggregation at the center, and ‘uniform’ for low-concentration distribution. We have classified these patterns in the table based on visual analysis of the distribution images. Notably, longer and more cohesive Nups, such as Nup116 and Nup100 tend to aggregate at the center, while less cohesive Nups, like Nup156 and Nup60 exhibit a uniform distribution. Shorter Nups, including Nup49, Nup57, and Nup42, predominantly cluster around the pore walls, indicating a correlation between Nup size, cohesiveness, and their preferential location within the pore.

**Table 5.** Distribution of yeast FG-Nups within the pore geometry. Compiled data from three studies highlight how different types of FG-Nups with specific amino acid ranges (AAs) are distributed within the NPC. The distribution patterns are classified into three categories: ‘wall’ (aggregation near the pore walls), ‘center’ (aggregation at the pore’s center), and ‘uniform’ (even distribution without aggregation).

FG-Nup	Ghavami et al. [24]		Huang et al. [54]		Peyro et al. [55]	
	AAs	Distribution	AAs	Distribution	AAs	Distribution
Nup116	1-726	wall	1-966	center	1-965	center
Nup100	1-816	wall	1-800	center	1-815	center
Nup145	1-896	center	1-426	wall	1-458	wall
Nup49	1-472	wall	1-270	wall	1-269	wall
Nup57	1-541	wall	1-287	wall	1–286	wall
Nup42	1-430	wall	1-382	wall	1–430	wall
Nup1	1-934	center	201-1076	center	1–1076	center
Nsp1	1-620	uniform	1-601	center	1–636	center
Nup159	390-1460	uniform	388-1082	uniform	382–1116	uniform
Nup60	1-539	uniform	351-539	wall	1–539	uniform
Nup2	-	-	1-720	uniform	-	-

The impact of parameter variations on FG-Nups conformation was explored through several coarse-grained molecular dynamics studies. Pulupa et al. [56] analyzed the dynamics within the yeast NPC and found that increasing the off-rate of the FG-FG interactions shifted the distribution pattern from ‘center’ to ‘uniform’. A similar transition was noted by Ananth et al. [57], who studied the system with a single FG-Nup of Nsp1. They observed that substituting the phenylalanine in FG repeats with serine resulted in a change from ‘center’ to ‘uniform’ distribution. These observations suggest that reducing the cohesiveness of FG-Nups facilitates the dispersion of central aggregates, aligning with predictions for homopolymers [52]. Further supporting this notion, Fragasso et al. [58] examined a pore containing an artificial FG-Nup named NupX. They observed a reduction in the central aggregation density upon increasing the grafting distance and thus decreasing the grafting density, findings that also mirror the homopolymer scenario [52].

Experimentally assessing the FG-Nups distribution within the NPC presents significant challenges due to the pore’s diminutive size (40–60 nm in diameter) and the dynamic nature of FG-Nups. The most direct observation to date has been achieved through high-speed atomic force microscopy (AFM). Fisher et al. [59] used DNA origami techniques to craft an artificial nanopore of 40 nm in diameter with FG-Nups (Nup100 and Nsp1) anchored to its inner surface. High-speed AFM was then employed to examine the pore’s surface geometry filled with FG-Nups [60]. Their observations revealed condensates of FG-Nups at the pore’s center, aligning with simulation predictions. Notably, the condensate was more pronounced for Nup100 compared to Nsp1. They also noted that the denser regions of FG-Nups shifted over time, highlighting the dynamic nature of FG-Nups within the NPC.

## Interactions with transport receptors

For small cargo sizes, with sizes up to 30–40 kDa, these can move through the NPC using simple diffusion, but for larger cargoes require the binding with nuclear transport proteins or receptors (NTRs) in order to make it through the NPC [47]. In vertebrates, these nuclear transport proteins are known as importins or transportins, and they are key in the transport of larger molecules into the nucleus, especially since it requires no energy for the transport [47], and many studies have been done on importins and transportins.

The main mechanism through which the NTRs make it through the NPC is by binding with the FG-Nups in the intrinsically disordered domain in the center of the NPC [61]. The FG repeats in the FG-Nups have been observed to keep the F residues exposed to the surrounding solvent, even though it is hydrophobic, which allows it to be ready to be bound to the cargo [62]. The NTR also contains important structural details that aid with binding. NTRs have been observed to have hydrophobic motifs (especially sequences of FG amino acids) surrounded by hydrophilic amino acids, and the amount of the hydrophobic region that is exposed actually fluctuates as a result to what environment that it resides in, meaning that a larger portion of the hydrophobic region is exposed in more hydrophobic environments, such as the NPC transport channel [63,64].

Results from X-ray crystallography as well as molecular dynamics simulations both suggest that the FG sequences from the FG-Nups bind with the hydrophobic pockets in NTR [65], such as the FG motifs as observed from and NTR named Importin-β, and the plasticity of the NTR mentioned earlier can actually control the strength of the binding interaction between the NTR and the NPC [61]

However, it is not enough for the cargo molecule to have these NTRs attached to it, there are many other factors that play into the penetration of the NPC, since this is all a game of free energy. If the free energy is negative, then the cargo will likely penetrate through, but if it is not, its transport will be opposed exponentially [47,66]. When a molecule enters the NPC, there is a certain tradeoff that the NPC-cargo system must face. The FG-Nups chains are disordered and dynamic, thus having a high entropy, and if the cargo molecules binds to the FG-Nups, it can change this conformation, lowering the entropy of the system by locking these polymer chains in place [47]. However, by binding with the FG-Nups, the cargo and system get an enthalpic gain through these interactions [47]. So, essentially, these cargo molecules would want to bind with the FG-Nups, but not so much so that it causes too much conformational change in the FG-Nups and restricting the conformational ensembles of the polymer chains, thereby lowering the permeability of the barrier [47].

In fact, the dynamic nature of the FG-Nups seems to play quite an essential role in the permeability of the membrane, especially since it’s been observed that even while the FG-Nups is bound to the NTRs, it still exhibits a dynamic nature, with many degrees of motion [61], which agrees with what was observed in terms of the free energy change. Not only can the bound FG-motif move around when it’s bound, but it can also skim out of the hydrophobic pocket in the NTR, and then either move back into the pocket or completely desert the cargo [61]. From this, the ‘slide and exchange mechanism’ was proposed, in which when one FG-motif moves out of a hydrophobic pocket in the cargo, it can be easily replaced or ‘exchanged’ by another FG-motif [61].

Another important aspect of the interactions between NTRs and FG-Nups is their multivalency. Due to the multiple-binding sites on both NTRs and FG-Nups, these molecules can engage in numerous simultaneous interactions. This multivalency enhances the affinity and specificity of these interactions, which is crucial for the selective and efficient transport through the NPC. Hayama et al. quantified the thermodynamic characteristics of the multivalent interactions using NMR and isothermal titration calorimetry [67]. They discovered that the affinity of individual FG-motifs with NTR binding sites is low enough to maintain the dynamic nature of FG-Nups. However, the multivalent nature of the binding increases the global avidity, resulting in enhanced selectivity in NTR uptake.

A recent computational study by Davis et al. further suggested that this global avidity changes sensitively to the distribution of the binding sites [68]. Their results showed that a more clustered arrangement of FG-repeats increases the probability of simultaneous multi-point interactions with NTRs, leading to higher avidity of the NTR-FG-Nups interactions. Similarly, higher densities of binding sites on NTRs improved the overall avidity. This result is consistent with the implications of other studies [66,68–70], highlighting the importance of spatial organization in modulating the strength and specificity of molecular interactions within the NPC.

## Challenges and limitations

Due to both the size and the intrinsically disordered nature of its nucleoporins, there are several challenges that are faced when attempting to observe it’s function or even model the NPC.

In terms of computational modeling, one of the main challenges faced when modeling it is that the interactions involved with the NPC and the cargo molecule can be very complex, and consequently resulting in computationally expensive models [71]. Furthermore, since the complex is made up of hundreds of proteins (around 600), that also proves to make modeling computationally difficult [71]. In order to effectively use computational modeling for the NPC, several simplifications to the complex must be made [71]. Most prominently, the trade off seems to be made between accounting for structural rearrangement of the complex and intermolecular reactions between the cargo molecule and FG-Nups, so MD models will typically choose to precisely model one while simplifying the other, and this is so that the model isn’t too computationally expensive and can be reasonably executed [71].

The sheer size and dynamicity of the NPC doesn’t only contribute to the difficulty in computationally modeling it, but it also has made imaging the structure of the complex difficult as well [72]. No one technique can quite give the full picture of the structure or function of the complex, and so in order to accurately piece this together, the integration of several techniques, such as electron microscopy, atomic force microscopy, crystallographic spectroscopy, and many others need to be combined in order to get a sense of how the NPC is structured and how it functions [72]. Piecing out the information obtained by each technique, however, has not been consistent amongst researchers, and there are multiple interpretations for the arrangement of the complex [72].

These limitations have made it difficult to both theoretically and experimentally observe the NPC. Despite this, there have been many advancements that helped understand the NPC while working with these limitations. For example, in the case of imaging, although each technique only gives us one vantage point of the complex, each one of these techniques have been refined such that they each give us ‘an exceptional view’ from their perspective [72]. In fact, computational techniques such as the jigsaw approach to modeling the FG-Nups, take the known and partially determined data about the structure of the NPC and affinities of the FG-Nups to try to fit a model to it [71].

## Conclusion and perspective

In this review, we have explored the physical properties of FG-Nups through the lens of polymer physics. Despite the challenges posed by the intrinsically disordered nature of FG-Nups, applying polymer physics principles has provided critical insights into understanding their behavior. Theoretical and computational studies employing the homopolymer approximation have delineated the morphological characteristics of FG-Nups with a limited set of parameters. These principles help to overcome the inherent disorder by simplifying the complex behaviors into more manageable models. A substantial body of experimental and simulation research has tested these models against FG-Nups. In many instances, the behavior of FG-Nups aligns well with predictions made under the homopolymer scenario, streamlining our understanding and making it more manageable.

To further solidify our understanding of FG-Nups, future studies should aim to uncover the effects of heterogeneous residual patterns and the dynamic features of FG-Nups. While the homopolymer approximation has provided a systematic view of FG-Nup conformation, FG-Nups inherently contain heterogeneity in their sequences, which potentially introduces variation in their conformational characteristics [73]. The dynamics of FG-Nups, such as their relaxation time and on-off kinetics between FG-motifs, are additional crucial features not explored in this review. These dynamics significantly influence the motion of FG-Nups and transport dynamics within the NPC [74,75].

The heterogeneity of FG-Nups is most evident in their residual sequences. Instead of being entirely homogeneous, FG-Nups contain hydrophobic FG-motifs distributed over their sequences, interspersed with hydrophilic residues [76,77]. Recent studies have shown that the spacing distance between these motifs affects the conformation and dynamics of FG-Nups [68,73]. Moreover, charged residues within FG-Nups are believed to alter the energy landscape within the NPC [78]. Bioinformatic studies have revealed specific patterns of charged residues in FG-Nup sequences [55,79]. These findings underscore the importance of sequence heterogeneity in FG-Nups. Investigating the effects of the heterogeneity, which cannot be fully captured by the homopolymer approximation, is a critical task for future research.

While we have an expanding collection of data regarding the conformational features of FG-Nups, our understanding of their dynamics remains limited. This limitation arises from the gap between the rapid dynamics of FG-Nups and the timescale of the experimental observation. For example, the spatiotemporal dynamics of FG-Nups assembly within the NPC can be observed using high-speed atomic force microscopy, which captures the movement of FG-Nups at a 100-millisecond frame rate [80,81]. However, the resealing of the nanopore occurs on the timescale of microseconds [75], which cannot be measured by current technology. Since the dynamics of FG-Nups are crucial for the regulation of molecular transport, it is important that future technological advancements, as well as the use of simulations, address this gap.

The advancement in polymer physics theory and experimental technologies provides promising avenues to further investigate these topics. Theoretical descriptions of polymer diffusion [82–84] and particle diffusion [85–89] within associative polymer networks hold potential applications for FG-Nups systems. Experimental advancements in single-molecule tracking techniques enable the analysis of nanoparticle diffusion within FG-Nup networks [16,90–92]. High-speed atomic force microscopy continues to be a vital tool for observing the morphological dynamics of FG-Nups assembly in a pore geometry [80,81]. Recent successes in engineering artificial nanopores with tethered FG-Nups present opportunities to design precisely controlled experimental environments that mimic NPCs. All these technologies have the potential to significantly enhance our ability to study the structure and dynamics of FG-Nups as well as nanoparticle diffusion within FG-Nups networks.

Our ultimate goal is to unravel the selective transport mechanism of the NPC. Revealing the physical characteristics of FG-Nups is the first step toward addressing this issue. Using polymer physics and the homopolymer approximation, we have gained a better understanding of the conformational characteristics of FG-Nups, as summarized in this review. By expanding this knowledge to include heteropolymer scenarios and uncovering the dynamics of FG-Nups, we will be able to achieve a comprehensive understanding of how they behave within the NPC. This knowledge could potentially explain how FG-Nups control the diffusive motion of molecules within the NPC. As we continue to gain insights into the physical features of FG-Nups, we move closer to unlocking the mechanism of selective transport within the NPC.

## Supplementary Material

Nucleus_Clean.pdf

## Data Availability

Data sharing is not applicable to this article as no new data were created or analyzed in this study.
